# The Cure of Disease

**Published:** 1886-12

**Authors:** Henry Kiddle


### THE CURE OF DISEASE.

BY PROFESSOR HENRY KIDDLE.

Are there perfectly reliable remedies for disease? The professional *medicus* will answer—yes; if you have the knowledge and experience requisite for the discovery of those things which nature supplies for the purpose, and skill sufficient to apply them; and every M. D., who has graduated from a medical college and has been duly licensed, by the authority of the legislature to treat the diseased, professes to posess, at least approximately, that knowledge and skill. Nevertheless, all of them have many patients who die under treatment, leaving the poor doctor the only consolation (usually quite adequate), that the patient "died according to the books." What right, indeed, had he to live who could so obstinately resist the duly authorized treatment, and such profound knowledge and skill? Fortunately, or unfortunately, no "crowner's quest" is ever required to pass upon that question.

Some physicians rely very much upon drugs, and attack nature, by pill and potion, at every vulnerable point, as if she, and not the disease, were to be overcome. Thus they often inflict many diseases in order to cure the one they are *scientifically* grappling with, on the principle of the doctor who, if he could not cure the patient's malady, could give him fits, and "he was death on fits."

On the other hand, the best physicians seek to interfere as little as possible with the curatic operations of Nature, endeavoring only to supply conditions for these, and to remove obstructions to them. These realize that there is a *vis medicatrix naturae*, which, if the disturbance can be removed, or the lesion repaired, will infallibly do this, unless prevented by counteracting agencies or untoward conditions. Such physicians appreciate the statement of the physiologist quoted by D'Alembert, who said satirically:

"Nature is fighting with a disease; and a blind man armed with a club, who calls himself a physician, comes to settle the contention. He first tries to make peace; and when he fails to do that, he lifts his club and strikes at random. If he hits the disease, he kills it; but if he strikes nature, he kills nature."

This satire is, of course, more applicable to the Sangrados and empirics of a former period than to the practice of this time; but it is not yet wholly obsolete. Montaigne's practice is, on the principle above referred to, as wise and safe as it was in his own time. He says, in one of his inimitable essays :

"When I am sick, I hate physic more than when I am well, telling those who importune me to take it they must give me time to recover my strength first, so that I may be able to encounter and support the violence and danger of the potion. I am afraid lest, instead of assisting nature when she is struggling with the disease, I should aid her adversary, and thus give her more work to do."

With the same idea, Pliny said : "Every disease is either curable or incurable ; a man recovers of it or is killed by it ; both ways physic is to be rejected. If it be deadly, it cannot be cured ; if it be curable, nature will cure it without a physician."

These, doubtless, are extreme opinions ; but certainly, nature is a greater healer than any of the certificated physicians, or "regular practitioners," as they are called, who contend for orthodoxy in medicine even more vehemently than the "ordained clergy" hold to orthodoxy in theology. Their bigotry is intense, and often disastrous. Dr. Buchan, author of the well-known work, "Domestic Medicine," said :

"Very few of the valuable discoveries in Medicine have been made by physicians. They have in general, either been the effect of change or of necessity, and have been usually opposed by the Faculty, till every one else was convinced of their importance."

The allopathic practitioners neglect and despise, generally, the use of everything but drugs, in considerable quantity and potent external applications. They refuse to recognize the finer forces, such as animal magnetism, for example, which, subtle as they are, are really the most powerful in nature. Have not Hahnemann and his followers, the Homæopathists, demonstrated by their alternated medicines and infinitesmal doses, that the curative power of the drug depends upon its approximation in tenuity to those subtle forces, or agencies? The influence of light and color in what has been called *Chromopathy*, affords an illustration of this principle.

The morbific agencies with which we are surrounded, and from which we cannot wholly escape, are all of an exceedingly subtle nature, and most insidious on that account. These, it is obvious, must be combated by remedial agents that, without producing further disturbances, will enter into the very penetralia of physical life, and expel the intruder or neutralize his deadly work

To learn what these life-giving, life-supporting forces and agencies are the physiologist and physician need an acute, comprehensive, unprejudiced mind. Excessive attachment to preconceptions dims the intellectual vision, and bigotry destroys it ; and then we have nothing but the blind man and the club, with a license, by act of the legislature, to kill with impunity—at least to make the apothecary's business prosperous, if not the "funeral directors." Woe unto the so-called physician who, through inexcusable and fatal bigotry, shuts himself up to one system of pathological science, without regard to the accredited experience of others in other modes of treatment—who persuades himself, *a priori*, that all statements opposed to his preconceived ideas must necessarily be false. This error is as bad in medicine as it is in religion ; the one affecting the bodies, the other the souls of those who trust to such false guidance.

Most physicians are too materialistic to recognize any forces and agencies beyond those of gross matter. Like the celebrated Dr. Lawrence, they have no faith in the existence of the soul, because they have never been able to find it with the scalpel. Thus they exclude themselves from avenues to knowledge of vital importance both to themselves and their patients. Would they consent to study that vast body of facts and phenomena that show the interrelations of body and soul, or spirit, they would achieve a success far transcending that which is possible, under their present materialistic systems, even to the most skillful and experienced. Here is opened a vast field of research, which at the present time is expanding in extent and increasing in importance every day.

